# Depletion of *KNL2* Results in Altered Expression of Genes Involved in Regulation of the Cell Cycle, Transcription, and Development in *Arabidopsis*

**DOI:** 10.3390/ijms20225726

**Published:** 2019-11-15

**Authors:** Anastassia Boudichevskaia, Andreas Houben, Anne Fiebig, Klara Prochazkova, Ales Pecinka, Inna Lermontova

**Affiliations:** 1 Leibniz Institute of Plant Genetics and Crop Plant Research (IPK) Gatersleben, Corrensstrasse 3, D-06466 Seeland, Germany; houben@ipk-gatersleben.de (A.H.); fiebig@ipk-gatersleben.de (A.F.); 2 Institute of Experimental Botany, Czech Acad Sci, Centre of the Region Haná for Biotechnological and Agricultural Research (CRH), Šlechtitelů 31, CZ-77900 Olomouc, Czech Republic; prochazkova@ueb.cas.cz (K.P.); pecinka@ueb.cas.cz (A.P.); 3 Mendel Centre for Plant Genomics and Proteomics, CEITEC, Masaryk University, Brno CZ-62500, Czech Republic

**Keywords:** *Arabidopsis*, KNL2, kinetochores, RNA-seq, centromere

## Abstract

Centromeres contain specialized nucleosomes at which histone H3 is partially replaced by the centromeric histone H3 variant cenH3 that is required for the assembly, maintenance, and proper function of kinetochores during mitotic and meiotic divisions. Previously, we identified a KINETOCHORE NULL 2 (KNL2) of *Arabidopsis thaliana* that is involved in the licensing of centromeres for the cenH3 recruitment. We also demonstrated that a knockout mutant for KNL2 shows mitotic and meiotic defects, slower development, reduced growth rate, and fertility. To analyze an effect of *KNL2* mutation on global gene transcription of *Arabidopsis,* we performed RNA-sequencing experiments using seedling and flower bud tissues of *knl2* and wild-type plants. The transcriptome data analysis revealed a high number of differentially expressed genes (DEGs) in *knl2* plants. The set was enriched in genes involved in the regulation of the cell cycle, transcription, development, and DNA damage repair. In addition to comprehensive information regarding the effects of *KNL2* mutation on the global gene expression, physiological changes in plants are also presented, which provides an integrated understanding of the critical role played by KNL2 in plant growth and development.

## 1. Introduction

The centromeres are specialized chromosomal domains that are required for proper separation of chromosomes during mitosis and meiosis. The centromere is composed of centromeric DNA, often enriched in satellite repeats, and the large protein complex “kinetochore”. In centromeric nucleosomes of most eukaryotes, histone H3 is partially replaced by the centromeric histone H3 variant cenH3 (also known as CENP-A in mammals, CID in *Drosophila*, Cse4 in *Saccharomyces cerevisiae*, and Cnp1 in *Schizosaccharomyces pombe* [1]). Deposition of cenH3 at the centromeric region is a prerequisite of the correct assembly and function of the kinetochore complex. It depends on different cenH3 assembly factors and chaperones [2], the transcription of the centromeric repeats [3,4,5], and the epigenetic status of centromeric chromatin [6,7]. In mammals, the Mis18 complex composed of Mis18α, Mis18β, and Mis18-binding protein 1 (also known as KNL2) plays an important role in the licensing of centromeres for cenH3 recruitment [8,9]. The human Mis18 protein complex localizes to centromeres during late telophase and remains associated with the centromere during early G1 phase when new CENP-A is deposited [2,9,10]. It mediates the recruitment of the cenH3 chaperone Holliday junction recognition protein (HJURP) to endogenous centromeres [11,12]. Knockout of murine Mis18a is embryo lethal [7]. Cultured homozygous mutant embryos showed misaligned chromosomes, anaphase bridges, and lagging chromosomes [7].

Up to now, only the cenH3 assembly factor KNL2 has been identified and characterized in plants [13,14]. In contrast to the mammalian cenH3 assembly factor Mis18BP1, the *Arabidopsis* KNL2 protein is present at centromeres during all stages of the mitotic cell cycle, except from metaphase to mid-anaphase [13]. Knockout of *KNL2* in *Arabidopsis* resulted in a reduced amount of cenH3 at centromeres, mitotic and meiotic defects, decreased DNA methylation degree, and lowered growth rate and fertility [13].

All homologs of Mis18BP1 (KNL2) identified up to now contain the conserved SANTA domain [15] at the N-terminus. However, the functional role of this domain still remains obscure. It was suggested that it might be involved in protein–protein interactions due to the presence of many conserved hydrophobic residues. It was shown that an absence of the SANTA domain in *Arabidopsis* KNL2 does not disturb its centromeric localization. Recently, the conserved C-terminal CENPC-k motif [14,16] required for the targeting of Mis18BP1 (KNL2) to centromeres was identified [14,17,18]. It presents in the Mis18BP1 (KNL2) proteins of most eukaryotes excluding therian mammals and *Caenorhabditis elegans* [14,16]. *Arabidopsis* KNL2 binds centromeric repeat *pAL1* and non-centromeric DNA sequences *in vitro*, whereas *in vivo* it associates preferentially with the centromeric repeat *pAL1*. The level and function of the Mis18BP1 protein in human cell culture is regulated by SUMOylation [19], and its centromeric localization is controlled by the phosphorylation in a cell cycle-dependent manner [20]. Whether the KNL2 of plants is regulated in the similar way remains to be elucidated.

Although the Mis18 protein complex is important for the deposition of cenH3 to centromeres in different organisms, its mechanism of function remains to be elucidated in detail. For mammals, it was shown that an interaction of the Mis18 complex with the *de novo* DNA methyltransferases DNMT3A and DNMT3B is required for the regulation of the epigenetic status of centromeric DNA and subsequently the transcription of centromeric repeats [7]. A knockout of mammalian Mis18α resulted in reduced DNA methylation, altered histone modifications, and increased centromeric transcripts in cultured embryos [7]. However, it was not tested whether a knockout of Mis18 complex components has an effect on the methylation status of non-centromeric chromatin and on the expression of other repetitive or gene-coding chromosomal regions. For instance, knockout of *KNL2* resulted in decreased DNA methylation of the marker regions *MEA-ISR* and the *At-SN1* in *Arabidopsis* [13]. 

In the current study, we used an RNA-sequencing (RNA-seq) approach to address the question of whether the inactivation of KNL2 influences the genome-wide gene expression during seedling and flower bud stages in *Arabidopsis.* This analysis allowed the identification of highly differentially expressed genes (DEGs) in flower buds (*n* = 1861) and seedlings (*n* = 459) of the *knl2* mutant. Gene Ontology (GO) term enrichment analysis links the activity of KNL2 to centromere function, DNA repair, DNA methylation as well as regulation of transcription. The specific pattern of gene expression in response to the inactivation of the *KNL2* gene provides a resource for future functional studies to unravel the role of KNL2 in kinetochore assembly and function.

## 2. Results and Discussion

### 2.1. Loss of KNL2 Function Leads to Massive Transcriptional Changes

To understand the role of *KNL2* for the kinetochore function, mitotic and meiotic divisions, and subsequently for plant growth and development, we compared the transcriptomes from two tissue types, namely seedlings and flower buds, of wild-type and the *knl2* plants. The experiments in the current study were designed in a way to weaken factors that could introduce experimental noise and would diminish the biological relevance of the data. For the RNA-seq study, we compared control and mutant plants with the same genetic background (Columbia ecotype) and physical age by performing experiments under the same plant growth conditions. This allowed the identification of DEGs and enriched biological processes. At the same time, the information obtained is limited to a single snapshot of gene expression reflecting the physical age of plants. Further comparative experiments including more time points to study temporal gene expression profiles in the *knl2* mutant line would be desirable, such as the comparison of the transcriptomes of *knl2* and wild-type plants not of the same “physical” but “biological age”, since the *knl2* mutant plants showed a delay in growth and development [13].

**Figure 1:**
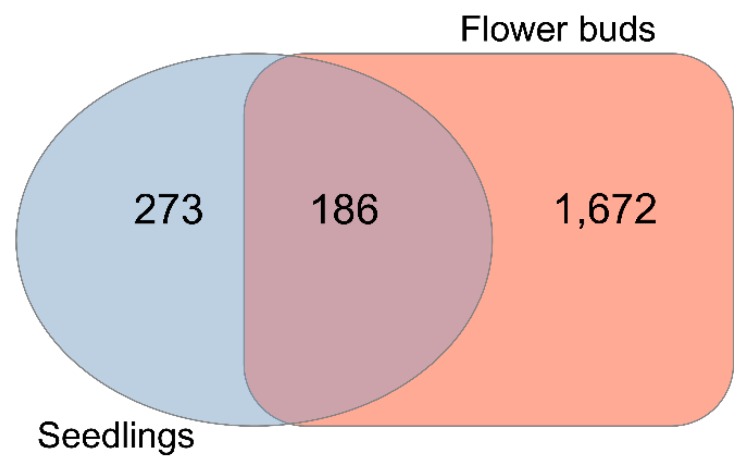
Differential gene expression. Venn diagram showing the number of highly differentially expressed genes (DEGs) (2-fold cutoff, false discovery rate (FDR) corrected *p*-value < 0.05) obtained by comparing *knl2* versus wild-type ecotype Columbia-0 (Col-0) genes in seedlings and *knl2* versus Col-0 genes in flower buds.

RNA-seq analysis based on DeSeq2 [21] identified 3261 genes in seedlings (Table S1, Supplementary Materials) that were altered in the *knl2* mutant line (adjusted *p*-value < 0.05). Among them, 459 genes (Figure 1) were highly differentially expressed (fold change (FC) cutoffs of ≥ 2).

In flower buds, more DEGs were identified in the *knl2* (4750 in total). Out of 1858 highly expressed genes (FC cutoffs of ≥ 2) (Figure 1), 1194 were highly upregulated and 664 were highly downregulated in the *knl2* mutant (adjusted *p*-value < 0.05). In both tissues, more genes were significantly upregulated than downregulated (Table S1).

In agreement with previously published data [13], which showed absence of the full-length *KNL2* transcript in the *knl2* plants, the mRNA level of *KNL2* was greatly reduced (log_2_ FC of −2.63 and −4.57) in seedling and flower bud tissues, respectively, of *knl2* plants, suggesting that the observed transcription responses are the result of *KNL2* depletion. 

To explore the distribution of DEGs and to identify the biological consequences of the *KNL2* inactivation, we used the GO::TermFinder [22]. Gene Ontology (GO) enrichment analysis for the “Biological process” was compared for downregulated and upregulated DEGs (adjusted *p*-value < 0.05) represented in seedlings and, separately, in flower buds. Detailed information about all overrepresented processes is given in a supporting information dataset (Dataset S1, Supplementary Materials). In this paper, we consider in more detail the categories associated with cell division, chromatin status, and plant growth and development. The results of the GO enrichment analysis can shed new light on how *KNL2* depletion leads to plant growth and development alterations. 

### 2.2. Knockout of KNL2 Impairs Expression of Genes Involved in Kinetochore Function

Kinetochores are responsible for the accurate segregation of chromosomes during mitosis and meiosis. Our RNA-seq analysis revealed GO terms related to physical events associated with both types of cell division, such as the “chromosome segregation”, “meiosis I”, “meiotic chromosome separation”, “mitotic metaphase plate congression”, “mitotic recombination”, and “attachment of mitotic spindle microtubules to kinetochore” in seedlings (Table 1). All DEGs representing these categories were downregulated in the *knl2* plants (Dataset S1, Tables S2 and S3).

GO terms characterizing the functions of kinetochores, like microtubule binding, chromosome movement, and checkpoint signaling were overrepresented in the downregulated gene list of *knl2* flower bud samples (Table 2).

For instance, reduced transcript levels were found for genes with known microtubule-associated functions [23] such as *KINESIN 5* (*ATK5*; AT4G05190), *TITAN1* (*TTN1*; AT3G60740)*,* and *HINKEL* (AT1G18370). The *CELL DIVISION CYCLE 20.1* (*CDC20.1*; AT4G33270), known for its critical role in the spindle assembly checkpoint-dependent meiotic chromosome segregation [24], was also significantly suppressed in the flower buds of *knl2* plants. Another example is the reduced expression of *BUB1* (AT2G20635), a protein kinase containing the Mad3-BUB1-I domain. Being together with CDC20.1 in the same protein–protein interaction network, BUB1 plays an important role in the assembly of the checkpoint proteins at the kinetochore [25]. In our RNA-seq study, it was highly downregulated in both types of tissue. Several other kinetochore associated genes, like *GAMMA-TUBULIN* (*TUBG1*; AT3G61650), *PLEIADE* (*PLE*; AT5G51600), and *AURORA KINASE 1* (AT4G32830) were downregulated in *knl2* flower buds. The expression of the inner kinetochore *CENP-C* (AT1G15660) was decreased in both seedlings and flower buds. Knockout of *KNL2* results in a reduced amount of *cenH3* transcripts of *Arabidopsis* [13]. This result was confirmed by RT-qPCR analysis of the samples used for the current RNA-seq study.

### 2.3. Reduced DNA Methylation in the knl2 Mutant Might Be Responsible for the Activation of a High Number of Transposons 

Knockout of *Arabidopsis KNL2* leads to a reduced level of DNA methylation [13]. In our RNA-seq study, a number of genes related to the DNA and histone methylation showed repressed differential expression in the *knl2* line (in both tissues). For example, histone H3 lysine 9 di-methyltransferase *SUVH4* (AT5G13960), *CHROMOMETHYLASE 2* (*CMT2*; AT4G19020), and *ARGONAUTE 6* (*AGO6*; AT2G32940) were found among differentially downregulated genes in the flower buds (adjusted *p*-value < 0.05), whereas *DEMETER-LIKE 1* (*DML1*; AT2G36490) showed reduced expression in both tissues used for analysis. 

**Figure 2:**
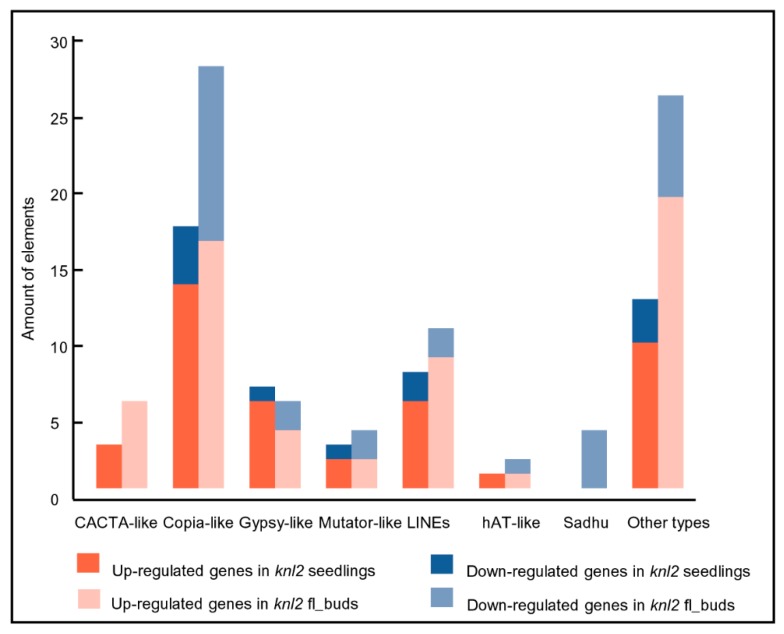
Transposable elements and their amount identified in the *knl2* mutant line. Information is given about transposons up- and downregulated in the *knl2* seedlings and *knl2* flower buds.

It is known that the activity of transposons is controlled epigenetically through DNA methylation and repressive histone marks (reviewed in [26,27,28]). It is likely that a reduced level of DNA methylation in the *knl2* mutant results in an altered expression of transposons. Indeed, a considerable number of transposable elements were differentially expressed in *knl2* seedlings (*n* = 52) and flower buds (*n* = 89). These elements belonged to the DNA transposons (*CACTA-like*, *hAT-like*, *Mutator-like*) and LTR retrotransposons (*Copia-like*, *Gypsy-like*). In both tissues, more transposons were upregulated in *knl2* plants (Figure 2). 

In contrast, the non-LTR retrotransposons *Sadhu 1-3* (AT3G44042), *Sadhu 3-1* (AT3G44042), *Sadhu 4-1* (AT5G28913), and *Sadhu 5-1* (AT4G01525) had decreased expression level in the *knl2* flower buds. Depression of *Sadhu 3-1* retroelement was also observed in the epigenetic mutant *suvh4* [29]. 

### 2.4. Altered Expression of the Root-related Genes Explains the Slow Root Growth of knl2 

**Figure 3:**
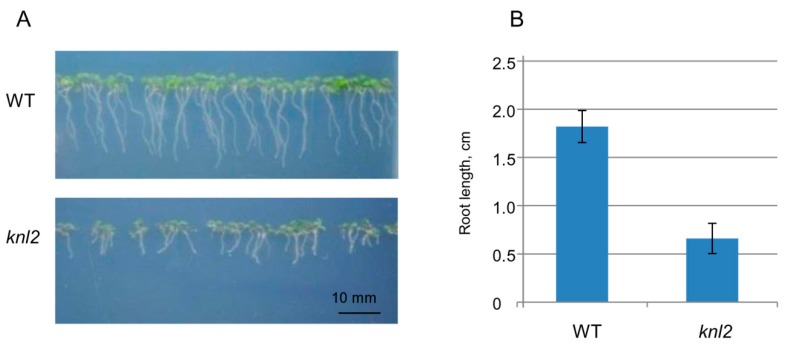
Effect of *KNL2* depletion on the root development. (**A**) Representative phenotypes of wild-type (WT) and *knl2* plants germinated and grown for seven days on ½ Murashige and Skoog (MS) medium. Bar = 10 mm. (**B**) Quantitative data for plants described in (**A**). Data are the means ± standard errors, *n* = 20. Student’s *t*-test, *p* < 0.01.

Root length was compared between seven-day-old control and the *knl2* mutant seedlings. The average root length of mutant seedlings (0.66 cm) was about 3 times shorter than that of the wild-type (1.82 cm) (Figure 3), and GO terms related to the root development such as “regulation of root development” and “primary root development” were highly overrepresented among the genes downregulated in the *knl2* seedlings (Table 1). 

We additionally tested all root-related genes available in TAIR for their overlap with the differentially expressed genes in the *knl2* seedlings. As a result, 119 differentially expressed genes were identified (Table S4, Supplementary Materials). Among them, 14 were highly suppressed (FC cutoffs of ≥ 2). The reduced transcript level was found for *SHORT AND SWOLLEN ROOT 1* (*SSR1*; AT5G02130). The *ssr1-2* plants show reduced root growth [30]. Other downregulated genes encoding the chloroplast/plastid localized GAPDH isoforms are *GAPCp1* (AT1G16300) and *GAPCp2* (AT1G79530). *gapcp* double mutants are characterized by arrested root development, dwarfism, and sterility [31]. The transcription factor *AGAMOUS-like 14*/*XAL2* (AT4G11880) preferentially expressed in roots is highly suppressed in *knl2* seedlings (log_2_ FC of −1.05, adjusted *p*-value 0.0001). Several studies demonstrated that *xal2* mutants have short roots [32,33]. In addition, we observed the reduced transcript level of *EMBRYO DEFECTIVE 2757* (*EMB2757*; AT4G29860) in *knl2* seedlings. Genetic analysis of *EMB2757* in *Arabidopsis* has demonstrated that mutations in this gene cause defects in both embryo and seedling development [34]. Upregulated differentially expressed genes in *knl2* seedlings can be further exemplified with *RAPID ALKALINIZATION FACTOR 23 (RALF23*; AT3G16570). Overexpression of *RALF23* leads to slower growing seedlings with roots that have reduced capacity to acidify the rhizosphere [35]. 

### 2.5. DNA Damage Repair Genes are Downregulated in the knl2 Plants

The genome stability is maintained by DNA damage responses. The failure to repair DNA damage leads to negative processes related to plant growth, reproduction, and even lethality. We found that genes representing the GO term “DNA repair” (26 genes) and related categories, such as “double-strand break repair via homologous recombination”, “non-recombinational repair”, and “DNA ligation involved in DNA repair” were overrepresented among *knl2* DEGs (Table 1). All genes of these categories were downregulated in the mutant. Among downregulated genes were the key players participating in the canonical nonhomologous end joining, such as *KU70* (AT1G16970), *KU80* (AT1G48050), and *LIGASE 4* (AT5G57160). It is known that mutations in these genes lead to increased sensitivity to double-strand DNA breaks (DSB)-inducing factors (reviewed in [36]). AT5G20850, encoding a DNA recombination and repair protein *RAD51*, is another example of a gene with a decreased expression level in *knl2* seedlings (log_2_ FC of −0.99, adjusted *p*-value 1.47 × 10^−6^). Loss of *RAD51* function does not affect the vegetative development of *Arabidopsis*, probably due to functional redundancy with other genes of the *RAD51* family, but is essential for meiotic repair of DSBs caused by *AtSPO11−1* [37]. In addition, the *RAD51-like* gene, *DISRUPTION OF MEIOTIC CONTROL 1* (*DMC1*; AT3G22880), known to promote interhomolog recombination was strongly downregulated in the *knl2* flower buds (log_2_ FC of −1.20, adjusted *p*-value 1.25 × 10^−8^). Interestingly, the mammalian kinetochore protein BUB1, playing an important role in chromosome segregation, is also known to participate in the DNA damage response [38]. In human and mouse cells, cenH3/CENP-A accumulates at DSBs together with CENP-N, CENP-T, and CENP-U [39]. Since the induction of DNA damage by radiation resulted in an increased expression of cenH3, the authors proposed a cenH3 function in DNA repair. Further research is needed to unravel the interrelationships between kinetochore genes and the DNA damage response.

**Figure 4:**
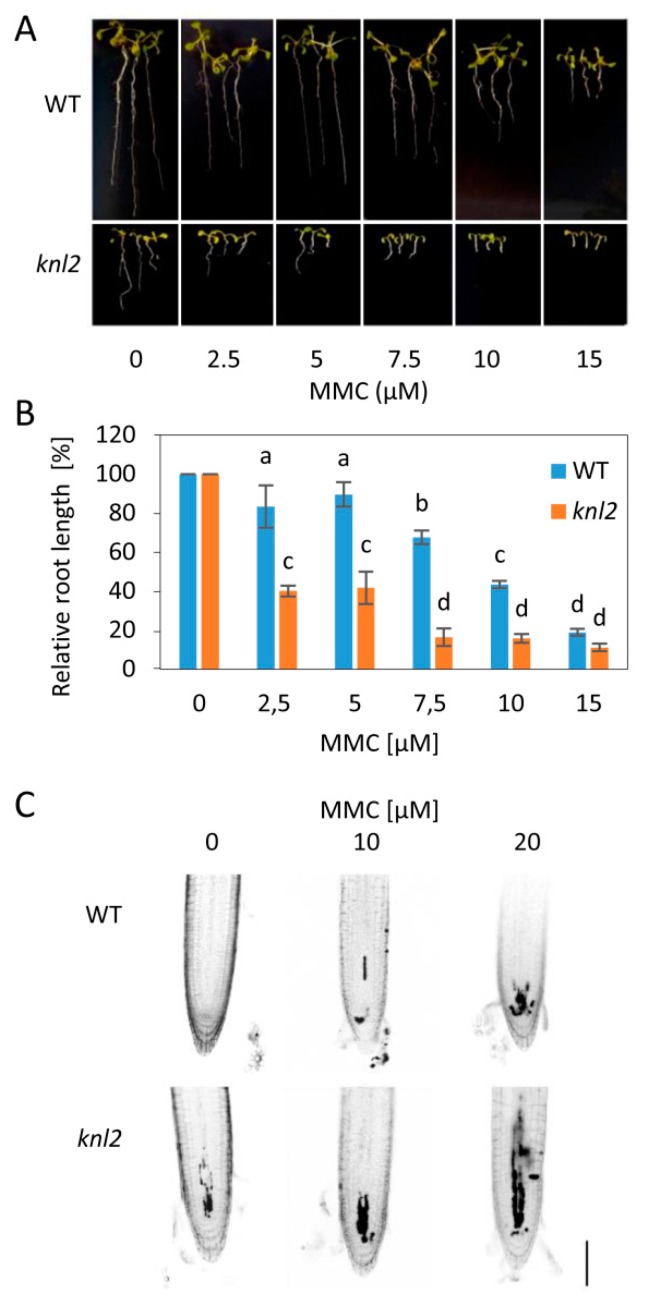
Sensitivity of *knl2* plants to DNA damage. (**A**) Representative phenotypes of wild-type (WT) and *knl2* plants grown for 14 days on mitomycin C (MMC)-containing media. Bar = 10 mm. (**B**) Quantitative data for plants grown as described in (**A**). Error bars represent the standard deviation between the means of three biological replicates, each represented by 15 to 20 plants. Letters above the bars indicate statistically significantly different groups in ANOVA (*p*-value ≤ 0.05) and post hoc Tukey‘s test. (**C**) Analysis of propidium iodide (PI) uptake (dark sectors) in roots of WT and *knl2* plants. Four-day-old plants were treated with the specified concentration of MMC for 24 h prior to analysis. Bar = 50 µm.

To test whether the altered mRNA levels are associated with changed capacity of *knl2* plants for DNA damage repair, we exposed wild-type and mutant plants to 10 µM DNA inter-strand crosslink inducer mitomycin C (MMC), 50 nM DNA strand breaker bleomycin, and 10 nM enzymatic DNA-protein crosslinker camptothecin (Figure S1, Supplementary Materials). This pilot experiment indicated sensitivity of *knl2* plants to MMC, but not other tested drugs (Figure 4). 

To validate this result, we treated *knl2* plants with a series of MMC concentrations (2.5 to 15 µM) and found significant reduction in their root growth compared to wild-type plants (Figure 4A,B). Next, we performed propidium iodide (PI) staining of root apices from plants treated for 24 h with 0, 10, and 20 µM MMC. Intense PI staining inside the cells indicates damaged plasma membranes, typical for stressed and dead cells. Under mock conditions, wild-type plant root apical meristems were intact, whereas the meristems of *knl2* plants showed few intensely stained cells. MMC treatment caused dose-dependent accumulation of intensely stained cells in the meristems of wild-type plants, but the increase in the meristem of *knl2* plants was more prominent. 

Based on this, we conclude that *knl2* plants fail to properly activate specific DNA damage repair factors, have reduced capacity for repair of DNA inter-strand crosslinks, and suffer from increased cellular damage.

### 2.6. Knockout of KNL2 Results in Deregulated Expression of a High Number of Transcription Factors

**Figure 5:**
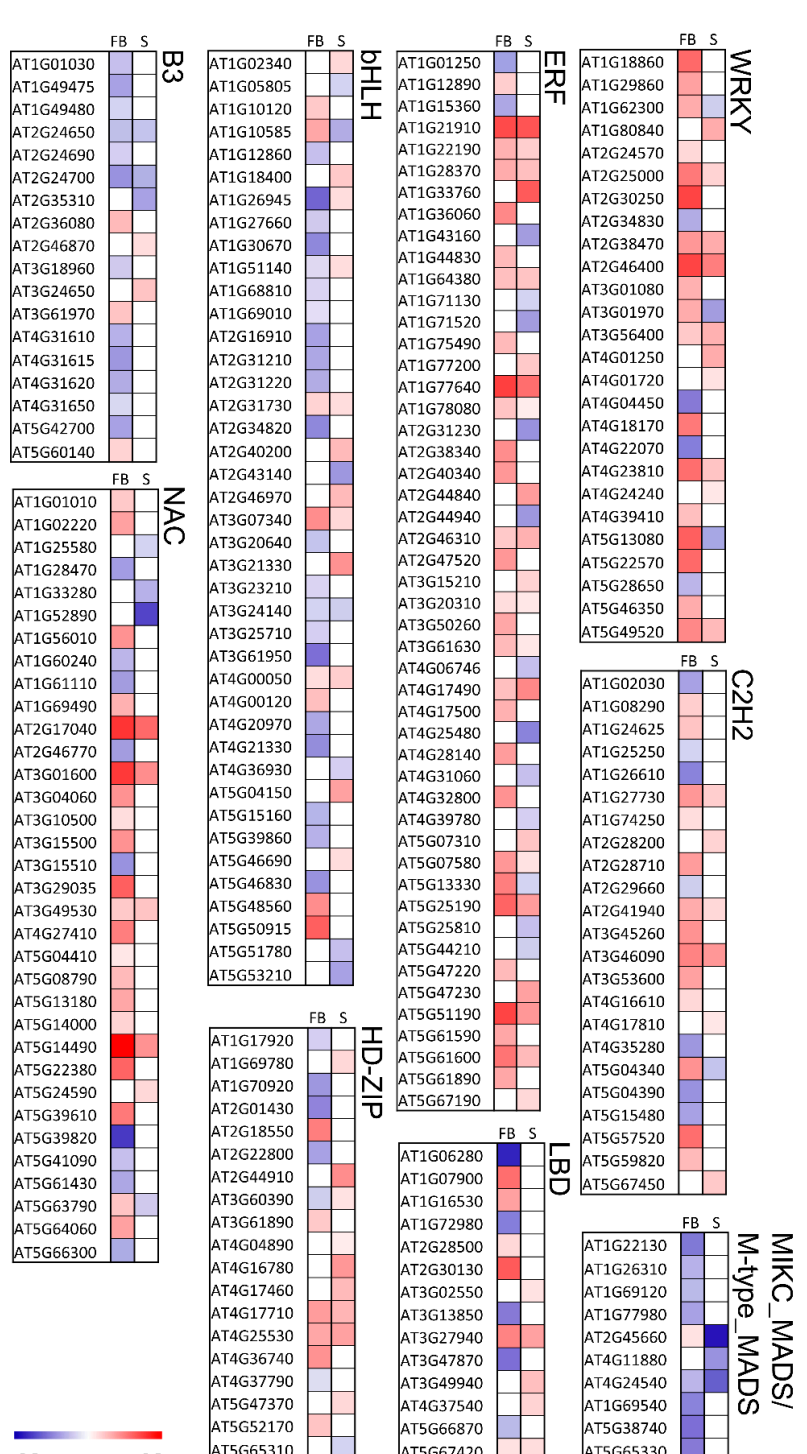
Differentially expressed genes encoding transcription factors in the *knl2* mutant line. Expression in *knl2* flower buds and *knl2* seedlings is depicted as F and S, respectively. Red and blue represent up- and downregulated differentially expressed genes, respectively. All values are log_2_ transformed. The gradient color illustrates the expression value.

We hypothesized that a large number of DEGs in the *knl2* mutant may be the result of deregulated expression of transcription factors. Therefore, the *knl2* mutant transcriptome was further screened for the presence of differentially expressed transcription factors (TFs). TFs were retrieved from The Plant TF database v.4 [40]. In *knl2* seedlings, 202 TFs were expressed (43 highly differentially expressed), whereas in *knl2* flower buds 330 TFs (137 highly differentially expressed) were detected. The classification of TFs is visualized in the heat map (Figure 5) and is given by the supporting information dataset (Dataset S2, Supplementary Materials). While most of the TF families showed a heterogeneous profile for the single TFs, some of the TF families behaved homogeneously (Figure 5). For example, the TF families B3 and MIKC_MADS and M-type_MADS mostly included downregulated genes in the *knl2* mutant, whereas the TF families ERF, NAC, and WRKY were enriched for upregulated genes. TF families in which many genes were highly differentially expressed were bHLH, C2H2, ERF, HD-ZIP, LBD, MIKC_MADS and M-type_MADS, NAC, and WRKY. The highest expression among TFs was observed in the NAC family. AT5G14490 encoding for NAC domain-containing protein 85 was highly upregulated in flower buds (log_2_ FC of 3.45, adjusted *p*-value 2.69 × 10^−12^). 

TFs with an altered expression in the *knl2* mutant are involved in different processes and provide insight into *Arabidopsis* development changes due to the inactivation of the kinetochore gene *KNL2*. There was a striking coordinated downregulation of genes representing the MADS-box family, which are the key regulators of seed and flower development (detailed description is presented below in 3.7). The upregulated TFs can be exemplified by the genes of the WRKY family. The TFs of this family play an important role in plant development and responses to environmental stress stimuli [41,42,43]. From 74 members of the WRKY TF family, 13 were differentially expressed in seedlings and 26 in the flower buds. The differentially expressed genes *WRKY33* (AT2G38470) and *WRKY46* (AT2G46400), highly upregulated in seedlings and flower buds, are known to play a role in heat stress responses. The expression of these genes was elevated in *MBF1c*-overexpressing plants, which showed enhanced tolerance to heat compared with wild-type plants [44]. *WRKY46* additionally regulates responses to other stresses in *Arabidopsis* [45,46,47]. 

Another TF family with overrepresented genes is ERF. The most highly activated genes in flower buds include *DREB19* (AT2G38340; log_2_ FC of 1.54, adjusted *p*-value 0.001), *DREB 26* (AT1G21910; log_2_ FC of 2.43, adjusted *p*-value 6.77 × 10^−7^), and *RAP2.6L* (AT5G13330; log_2_ FC of 1.75, adjusted *p*-value 3.54 × 10^−10^). A previous study of the *DREB19*, *DREB26*, and *RAP2.6L* effect in *Arabidopsis* demonstrated the participation of the genes in plant developmental processes as well as biotic and/or abiotic stress signaling [48].

The heat map also revealed genes that are differentially expressed in flower buds but not in seedlings and vice versa. We observed that 94 highly differentially expressed genes were present only in flower buds and 18 highly regulated genes were seedling-specific (Dataset S2). The transcriptional repressor of the RAV family *TEMPRANILLO 1* (*TEM1*), known to postpone floral induction [49], is another example of the highly upregulated gene present in the flower buds (log_2_ FC of 1.55).

### 2.7. Late Flowering of the knl2 Plants is Determined by the Altered Expression of Flowering Genes 

**Figure 6:**
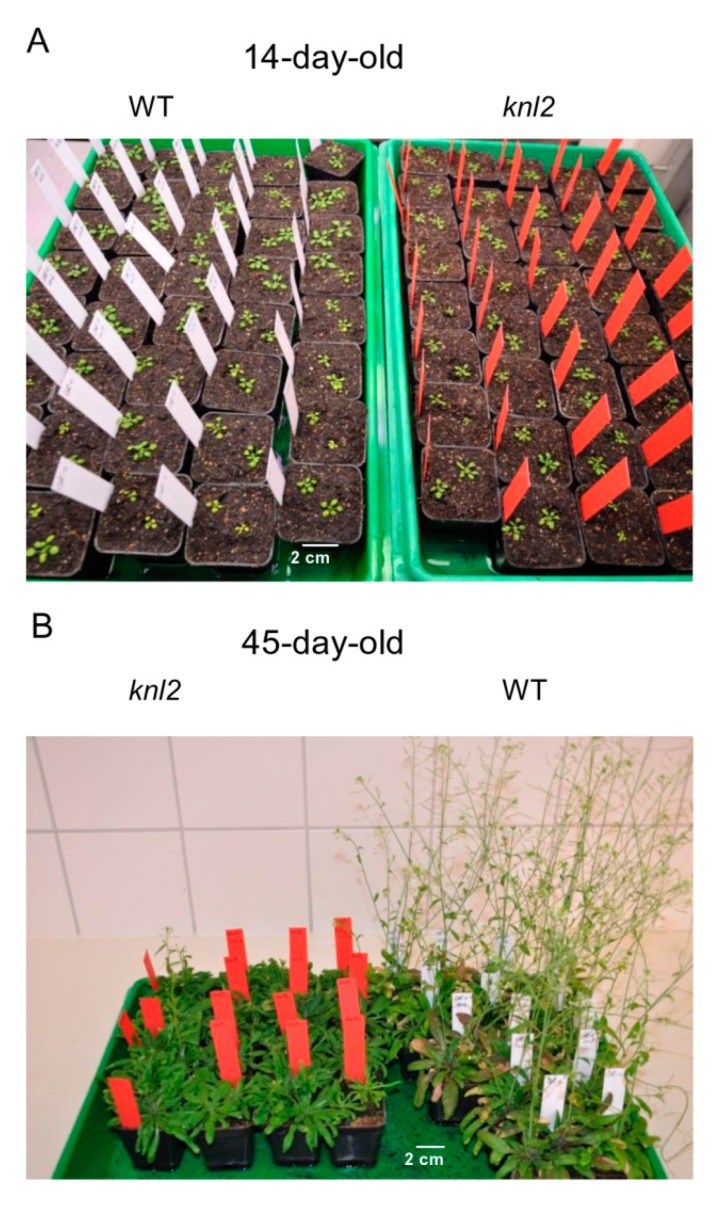
Effect of *KNL2* depletion on the flowering time. (**A**) At early growth stages, no obvious phenotypical differences between wild-type (WT) and the *knl2* mutant were observed. (**B**) The flowering time of the *knl2* mutant delayed by 10–14 days compared to *Arabidopsis* wild-type. Seeds of the *knl2* mutant and wild-type were germinated under short-day conditions, 8 h light/20° C and 16 h dark/18 °C, for two weeks and then plants were transferred to the cultivation room with long-day conditions, 16 h light/20 °C and 8 h dark/18 °C.

We observed that flowering of the *knl2* plants delays for 10–14 days compared to wild-type (Figure 6; Figure S2, Supplementary Materials). 

From this observation, we could identify a number of enriched GO terms related to the floral development in our RNA-seq data from *knl2* flower buds. These represent such GO categories as “reproduction”, “regulation of pollen tube growth”, “pollen germination”, “pollination”, and “pollen development” (Table 3). 

Genes representing these and related categories in Table 3 were downregulated in the *knl2* flower buds. Some of the genes belong to the MYB transcription factors and regulate the plant microgamete development. For instance, *AtMYB103* (AT1G63910), important for pollen development [50], is one of the highly suppressed flower bud genes in *knl2.* The expression of other prominent transcription factors like *AGAMOUS-LIKE 66* (*AGL66;* AT1G77980) and *AGL104* (AT1G22130) involved in pollen maturation and tube growth was significantly reduced. Adamczyk and Fernandez [51] demonstrated that double mutant plants *agl66 agl104* could produce pollen but had severely reduced fertility and aberrant pollen tube growth. Along with *AGL66* and *AGL104*, *AGL94* (AT1G69540) belongs to the MIKC * factors which are highly active in pollen as major regulators of pollen maturation programs [51,52]. These three genes were significantly downregulated in the *knl2* flower buds (adjusted *p*-value < 0.001). This finding might explain the reduced fertility of *knl2* plants in addition to the role of KNL2 in mitosis and meiosis [13]. 

The RNA-seq data analysis and observation of the delayed flowering prompted us to inspect the RNA-seq data of the *knl2* seedlings. We asked the questions of whether and, if so, how many flowering-related genes based on the FLOR-ID overlap with the differentially expressed genes in the *knl2* seedlings. From 306 genes, 36 genes could be identified (Dataset S3, Supplementary Materials). There was a striking coordinated downregulation of several prominent flowering-related genes. For example, the floral integrator *Flowering Locus T* (*FT*; AT1G65480), known to be expressed in leaves, was significantly downregulated in *knl2* seedlings. Its reduced expression is in correspondence with the downregulation of *SUPPRESSOR OF OVEREXPRESSION OF CONSTANS 1* (*SOC1*; AT2G45660) in the *knl2* seedlings (log_2_ FC of −2.24, adjusted *p*-value 7.72 × 10^−61^). It is known that flowering activator *SOC1* acts in a positive-feedback loop with *AGL24*, which is the important MIKC^c^-type transcription factor positively regulating flowering in *Arabidopsis* (reviewed in [53]). In our RNA-seq study, the *AGL24* showed reduced expression in both *knl2* seedlings and flower buds. 

*GIGANTEA* (*GI*; AT1G22770), an important gene in regulating photoperiodic flowering, had a reduced expression in the *knl2* mutant line compared to wild-type. Another regulator of transition to flowering *FKF1* (*ADO3;* AT1G68050) was highly downregulated in the *knl2* mutant line (log_2_ FC of −1.70, adjusted *p*-value 2.79 × 10^−17^). A member of the MADS-box family *XAANTAL2* (*XAL2/AGL14*; AT4G11880), which is a necessary and sufficient agent to induce flowering [54], showed decreased expression level in the *knl2* seedlings (log2 FC of −1.05, adjusted *p*-value 0.002).

The reduced activity of flower inductive pathway genes observed in the *knl2* seedlings might explain the delay in flower initiation, at least under long-day conditions.

### 2.8. Knockout of KNL2 Results in Altered Expression of Genes Controlling Seed Development

The loss-of-function *knl2* mutant showed reduced seed production [13]. We used the SeedGenes Project database including 481 genes in order to examine the overlap between these genes and the 4750 genes that we found to be significantly differentially expressed in response to *KNL2* inactivation. From these genes, 42 were differentially expressed in the *knl2* flower bud samples (Table S5, Supplementary Materials). Among downregulated genes, there are those whose disruption causes embryo abortion, such as *CYL 1* (AT5G13690), *EMB 1674* (AT1G58210), *EMB 2184* (AT1G75350), *EMB 3003* (AT1G34430), *EMB 3004* (AT3G06350), and *IMPL 2* (AT4G39120). Highly upregulated genes that include *HSP 17.4* (AT3G46230), *RLP37* (AT3G23110), and *NCED3* (AT3G14440) are known for their expression in seeds and response to abiotic stresses or defense.

### 2.9. Real-time Quantitative PCR Confirms RNA-seq Analysis

**Figure 7:**
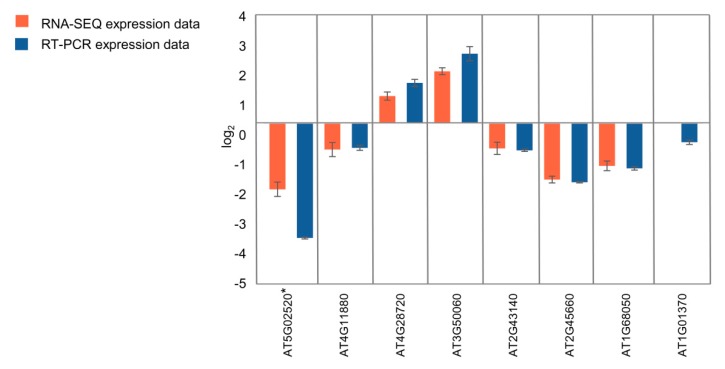
qRT–PCR validation of RNA-seq results. Eight genes were selected from the genes differentially expressed in seedlings according to the RNA-seq data analysis. Detailed annotation of the selected genes is presented in Table S2 (Supplementary Materials). *At5g02520 corresponds to *KNL2*. Error bars indicate the standard error of three biological replicates in qRT-PCR.

To validate the quality of the RNA-seq data by qRT-PCR, we selected eight genes differentially expressed in seedlings and 11 genes differentially expressed in flower buds (Figure 7; Figure S3, Supplementary Materials). In the case of seedlings, four genes involved in the regulation of root growth (AT4G11880, AT4G28720, AT3G50060, AT2G43140) and two genes involved in the regulation of flowering time (AT2G45660, AT1G68050) were selected for the analysis. In the case of flower buds, an expression of five genes involved in the regulation of flowering time (AT5G48560, AT2G40080, AT5G37770, AT1G10120, AT1G02580) and four genes involved in the regulation of seed development (AT2G01860, AT3G23110, AT5G13690, AT5G23940) was validated. *KNL2* (At5g02520) and *cenH3* (At1g01370) genes were included for the analysis in both types of tissues. Our qRT-PCR confirmed the extreme downregulation of *KNL2* gene expression in the mutant line. All RNA-seq-based determined up- and downregulated genes showed the same qualitative response in both seedlings and flower buds based on qRT-PCR (correlation coefficients between qRT-PCR and RNA-seq data of 0.996 and 0.974, respectively). 

### 2.10. Conclusions

KNL2 of *Arabidopsis* is involved in regulating the centromeric loading of cenH3, and its knockout results in mitotic and meiotic defects and reduced DNA methylation, followed by reduced growth rate and fertility compared to wild-type [13]. Our comparative RNA-seq analysis revealed that these abnormalities were associated with an altered expression of genes regulating corresponding processes. For instance, the reduced growth rate of roots and late flowering of the *knl2* mutant correlate with the altered expression of genes regulating root growth and flowering, respectively. We propose that KNL2 has an essential regulatory function in plant growth and development that is much broader than the regulation of centromeric localization of cenH3 only.

## 3. Materials and Methods

### 3.1. Plant Materials and Growth Conditions

The *A. thaliana knl2* mutant (SALK_039482) in Col-0 background was described previously [13]. Seeds of *A. thaliana* wild-type and *knl2* were germinated in Petri dishes on half strength Murashige and Skoog (MS) medium (Murashige and Skoog, 1962) for eight days. For harvesting of flower buds, populations of wild-type and *knl2* plants (30 plants per each population) were grown in soil until flowering. In both cases, plants were cultivated with a 16 h photoperiod (21 °C day/18 °C night), 70% relative humidity. Light irradiance at plant level was 130 µmol m^−2^ s^−1^. 

### 3.2. DNA Damage Sensitivity Assays

For the pilot root length assay experiment, plants were germinated and grown for seven days on ½ strength Murashige and Skoog medium containing 0.01% DMSO and different DNA damage inducers—mitomycin C (MMC) (Duchefa, Haarlem, The Netherlands), bleomycin (Calbiochem, San Diego, CA, USA), and camptothecin (Sigma-Aldrich, Saint Louis, MO, USA) with concentrations specified in the text. For the subsequent analysis, plants were germinated and grown for 14 days on media containing different concentrations of MMC (2.5, 5, 7.5, 10, and 15 µM from Sigma-Aldrich). For PI staining assay, plants were grown on solid ½ Murashige and Skoog medium for four days, then transferred to liquid ½ Murashige and Skoog medium containing 0, 10, and 20 µM MMC and grown for 24 h. Subsequently, the plants were stained with 10 μg/mL propidium iodide solution (Sigma-Aldrich) for 3 min, rinsed with tap water and analyzed using an AxioImager Z2 (Zeiss, Jena, Germany) microscope equipped with the DSD2 confocal module (Andor Technology, Belfast, Great Britain). Plants for all procedures were grown in a Percival growth chamber under long-day (16 h light) conditions and 21 °C.

### 3.3. RNA Isolation and Illumina Sequencing

Total RNA was extracted from the eight-day-old seedlings of *A. thaliana*. At least 60 seedlings (100 mg) were pooled to produce a biological replicate. For the RNA-seq analysis of flower buds, inflorescences were harvested from three individual plants for each sample (30 mg). Flower buds older than stage 12 and flowers [55] were removed. All tests were performed on three biological replicates per condition and genotype. Total RNA was isolated from seedlings and flower bud samples using the RNeasy Plant Mini Kit (Qiagen, Hilden, Germany) according to the manufacturer’s manual. The RNA preparations were checked for quality using a NanoDrop spectrophotometer and a 2100 Bioanalyzer (Agilent, Santa Clara, CA, USA). In total, 12 RNA samples (1 µg each) were provided to the IPK-Sequencing-Service (IPK, Gatersleben, Germany) for construction of cDNA libraries with the TruSeq RNA Sample Preparation Kit (Illumina, San Diego, CA, USA). The libraries were sequenced in a HiSeq2500 rapid 100-bp single-read run system After sequencing, the adapter sequences and the barcodes were removed. 

### 3.4. RNA-seq Data Processing 

Sequencing quality of the reads was examined by using FastQC Read Quality reports, Galaxy Version 0 0.72 [56]. At least 94% of the bases of each read in all samples possessed Illumina Quality >30 and no sequence flagged as poor quality was detected. 

Sequences were aligned against the *Arabidopsis* TAIR 10 genome assembly using HISAT2 Galaxy tool version 2.0.3. [57] with default settings. Read counts for each gene were quantified based on the BAM files produced with the HISAT2 by using the tool feature Counts, Galaxy Version 1.4.6.p5 [58]. The advanced setting parameters were strand specificity = “no”, GFF feature type filter = “exon”, and GFF gene identifier = “Parent”. Differentially expressed features were determined based on the feature Counts tables by applying the tool DESeq2, Galaxy Version 2.11.38 [21] with the setting parameter fit type = “parametric”. DESeq2 tested for differential expression based on a model using the negative binomial distribution. Differentially expressed genes were identified by comparison of two groups, namely, (1) a mutant line and the wild-type control, condition seedlings and (2) a mutant line and the wild-type control, condition flower buds.

The results of all statistical tests were adjusted for the multiple testing false discovery rate (FDR) with the Benjamini and Hochberg procedure [59]. A cutoff value of adjusted *p*-values equal to 0.05 was chosen as a threshold to identify significant differentially expressed genes.

### 3.5. Analysis of Differentially Expressed Genes (DEGs)

Functional characterization of the DEGs showing significant expression changes in response to *KNL2* depletion was done based on the TAIR10 annotation (https://www.arabidopsis.org/). Gene Ontologies were analyzed for term enrichment using the Generic Gene Ontology GO::TermFinder tool ([22]. The analysis was carried out using the Benjamini–Hochberg FDR with a filter *p*-value of < 0.05. 

Information about flowering-related genes was extracted from the Flowering Interactive Database [60]. The appearance of the transcription factors in the analyzed gene sets was confirmed with the Plant Transcription Factor Database v.4.0 [40]. Information about the genes essential for the *Arabidopsis* development from the SeedGenes Project [61] was used to find the corresponding genes among differentially expressed genes.

### 3.6. Gene Expression Validation by Reverse Transcription Quantitative PCR (RT-qPCR)

Total RNA extraction was performed as described above. The RNA was treated with DNase I (Ambion, Thermo Fisher Scientific, Waltham, MA, USA) according to the manufacturer’s protocol to eliminate any residual genomic DNA. Reverse transcription was performed using a first-strand cDNA synthesis kit, oligo dT (18-mer) primer (both Fermentas, Thermo Fisher Scientific, Waltham, MA, USA), and 2 μg of total RNA as a starting material. 

The gene-specific primers (Table S6, Supplementary Materials) were designed using the fully automated QuantPrime tool [62]. The amplification of the *UBQ10* (AT4G05320) reference gene [63] was used as an internal control to normalize the data.

Quantitative real-time measurements were performed using POWER SYBR Green Master Mix reagent in a QuantStudio 6 Flex system (Applied Biosystems, Thermo Fisher Scientific, Waltham, MA, USA), according to the manufacturer’s instructions. The cDNA equivalent to 40 ng of total RNA was used in a 10 µL PCR reaction.

The cycling conditions comprised 10 min polymerase activation at 95 °C and 40 cycles at 95 °C for 3 s and 60 °C for 30 s. Three biological replicates per genotype (wild-type and *knl2* mutant line) in both conditions (seedlings and flower buds) were tested. Each biological replicate was represented with three technical replicates, which were analyzed during the same run. Relative gene expression was calculated using the comparative method 2^−∆∆CT^ [64].

### 3.7. Data Availability

Raw reads are available under the accession number PRJEB32230 at http://www.ebi.ac.uk/ena/data/view/PRJEB32230.

## Figures and Tables

**Table 1:** Assignment of differentially expressed downregulated genes in *knl2* seedlings to different functional categories related to cell processes. Gene Ontologies were analyzed for term enrichment using the Generic Gene Ontology GO::TermFinder tool (cutoff of 5% false discovery rate (FDR)).

GO ID	Biological Process	*p*-Value	FDR Rate, %	Gene Count
GO:0051315	Attachment of mitotic spindle microtubules to kinetochore	0.002143	2.39	2
GO:0007049	Cell cycle	0.003143	2.94	41
GO:0000075	Cell cycle checkpoint	0.004300	3.40	5
GO:0022402	Cell cycle process	0.001001	0.92	30
GO:0007059	Chromosome segregation	0.007086	4.99	11
GO:0051304	Chromosome separation	0.001526	1.15	6
GO:0051103	DNA ligation involved in DNA repair	6.37 × 10^−5^	0.06	4
GO:0006310	DNA recombination	4.72 × 10^−6^	0.00	20
GO:0006281	DNA repair	0.000745	0.79	26
GO:0006302	Double-strand break repair via	1.79 × 10^−5^	0.02	15
	homologous recombination			
GO:0006303	Double-strand break repair via	0.000127	0.15	5
	nonhomologous end joining			
GO:0035825	Homologous recombination	0.001124	0.93	8
GO:0007127	Meiosis I	0.001483	1.10	9
GO:0051307	Meiotic chromosome separation	0.001680	1.31	4
GO:0051310	Metaphase plate congression	0.002143	2.40	2
GO:0007080	Mitotic metaphase plate congression	0.002143	2.44	2
GO:0006312	Mitotic recombination	0.000820	0.82	5
GO:0045786	Negative regulation of cell cycle	0.005582	4.02	7
GO:0000726	Non-recombinational repair	0.000127	0.15	5
GO:0007131	Reciprocal meiotic recombination	0.001124	0.94	8
GO:0071156	Regulation of cell cycle arrest	0.006230	4.82	2

**Table 2:** Assignment of differentially expressed downregulated genes in *knl2* flower buds to different functional categories related to cell processes. Gene Ontologies were analyzed for term enrichment using the Generic Gene Ontology GO::TermFinder tool (cutoff of 5% FDR).

GO ID	Biological Process	*p*-Value	FDR Rate, %	Gene Count
GO:0007049	Cell cycle	3.13 × 10^−9^	0.00	83
GO:0048468	Cell development	3.30 × 10^−8^	0.00	56
GO:0030154	Cell differentiation	2.36 × 10−^5^	0.08	95
GO:0051301	Cell division	5.97 × 10^−7^	0.00	54
GO:0016049	Cell growth	3.06 × 10^−6^	0.05	62
GO:0032989	Cellular component morphogenesis	2.59 × 10^−8^	0.00	72
GO:0016043	Cellular component organization	2.03 × 10^−8^	0.00	261
GO:0070192	Chromosome organization	0.000320	0.52	11
	involved in meiotic cell cycle			
GO:0007059	Chromosome segregation	0.001374	1.31	17
GO:0000910	Cytokinesis	3.63 × 10^−5^	0.15	21
GO:0007010	Cytoskeleton organization	6.02 × 10^−6^	0.04	33
GO:0061640	Cytoskeleton-dependent cytokinesis	0.000676	0.81	17
GO:0000086	G2/M transition of mitotic cell cycle	0.000205	0.41	7
GO:0048229	Gametophyte development	3.69 × 10^−9^	0.00	70
GO:0045143	Homologous chromosome segregation	5.80 × 10^−5^	0.18	10
GO:0035825	Homologous recombination	2.30 × 10^−5^	0.09	13
GO:0007127	Meiosis I	2.23 × 10^−5^	0,09	15
GO:0061982	Meiosis I cell cycle process	7.78 × 10^−6^	0.08	16
GO:0051321	Meiotic cell cycle	4.41 × 10^−5^	0.15	27
GO:0045132	Meiotic chromosome segregation	1.62 × 10^−5^	0.10	14
GO:0051307	Meiotic chromosome separation	0.001212	1.24	5
GO:0140013	Meiotic nuclear division	1.12 × 10^−5^	0.07	20
GO:0000226	Microtubule cytoskeleton organization	0.000596	0.78	17
GO:0007018	Microtubule-based movement	0.000235	0.44	10
GO:0000278	Mitotic cell cycle	5.20 × 10^−5^	0.19	36
GO:0000281	Mitotic cytokinesis	0.001316	1.29	16
GO:0006312	Mitotic recombination	0.001079	1,20	6
GO:0098813	Nuclear chromosome segregation	0.000706	0.85	16
GO:0000280	Nuclear division	0.000107	0.20	24
GO:0007131	Reciprocal meiotic recombination	2.30 × 10^−5^	0.09	13
GO:0051726	Regulation of cell cycle	0.000251	0.45	32
GO:0007129	Synapsis	7.30 × 10^−5^	0.19	9

**Table 3:** Assignment of genes differentially expressed in *knl2* flower buds to development-related functional categories based on GO::TermFinder. Genes downregulated in the *knl2* flower buds were included into the GO enrichment analysis (cutoff of 5% FDR).

GO ID	Biological Process	*p*-Value	FDR Rate, %	Gene Count
GO:0048653	Anther development	0.000711	0.84	13
GO:0048589	Developmental growth	0.004788	4.32	54
GO:0060560	Developmental growth involved	2.06 × 10^−5^	0.10	51
	in morphogenesis			
GO:0044703	Multi-organism reproductive process	0.002391	2.32	26
GO:0009555	Pollen development	4.10 × 10^−11^	0.00	61
GO:0010584	Pollen exine formation	2.12 × 10^−5^	0.09	10
GO:0009846	Pollen germination	0.000968	1.15	13
GO:0048868	Pollen tube development	1.57 × 10^−6^	0.00	36
GO:0009860	Pollen tube growth	7.09 × 10^−9^	0.00	34
GO:0009856	Pollination	2.44 × 10^−5^	0.08	44
GO:0010769	Regulation of cell morphogenesis involved	0.001673	1.84	9
	in differentiation			
GO:0080092	Regulation of pollen tube growth	0.000708	0.85	9
GO:0000003	Reproduction	3.55 × 10^−4^	0.50	189
GO:0048443	Stamen development	0.002527	2.46	15

## Supplementary Materials

Supplementary materials can be found at https://www.mdpi.com/1422-0067/20/22/5726/s1. Figure S1. *knl2* sensitivity to DNA damaging agents. The plants were grown for seven days on media containing specified drugs. Figure S2. Effect of *KNL2* depletion on the flowering time.The flowering time of the *knl2* mutant delayed by 10–14 days compared to *Arabidopsis* wild-type. Seeds of the *knl2* mutant and wild-type were germinated under short-day conditions, 8 h light/20° C and 16 h dark/18 °C, for two weeks and then plants were transferred to the cultivation room with long-day conditions, 16 h light/20° C and 8 h dark/18 °C. To demonstrate differences in flowering time between the *knl2* mutant and wild-type, images of plant populations were taken with different time intervals. At early growth stages (14-day-old plants), no obvious phenotypical differences between the Col wild-type and the *knl2* mutant were observed. Figure S3. qRT–PCR validation of RNA-seq results. Eleven genes were selected from the genes differentially expressed in flower buds according to the RNA-seq data analysis. Detailed annotation of the selected genes is presented in Table S2. *At5g02520 corresponds to *KNL2*. Error bars indicate the standard error of three biological replicates in qRT-PCR. Table S1. Results of the RNA-seq study based on the DeSeq2 in Col and the *knl2* mutant line. Conditions: seedlings and flower buds. Table S2. Differentially expressed genes in *knl2* seedlings and their annotation. Table S3. Differentially expressed genes in *knl2* flower buds and their annotation. Table S4. Identification of root-related genes in *knl2* seedlings. Table S5. Overlap between seed-related genes from the SeedGenes Project DB and differentially expressed genes in *knl2* flower buds. Table S6. Selected genes and their primers used for qRT-PCR. Dataset S1. GO terms overrepresented in the *knl2* mutant line. Conditions: seedlings and flower buds. Dataset S2. Overlap between TF from The Plant TF database v.4 and differentially expressed genes in the *knl2* mutant line. Dataset S3. Overlap between flowering-related genes from the database FLOR-ID and differentially expressed genes in the *knl2* mutant line.
