# Supplementary material for: Depletion of KNL2 Results in Altered Expression of Genes Involved in Regulation of the Cell Cycle, Transcription, and Development in Arabidopsis
Source: Int J Mol Sci. 2019 Nov 15;20(22):5726. doi: 10.3390/ijms20225726 (PMC6888302; doi:10.3390/ijms20225726)
Supplement: Supplementary file 1 [file ijms-20-05726-s001.zip › supplemental/Supplemental Figure 3.pptx]

## Slide 1
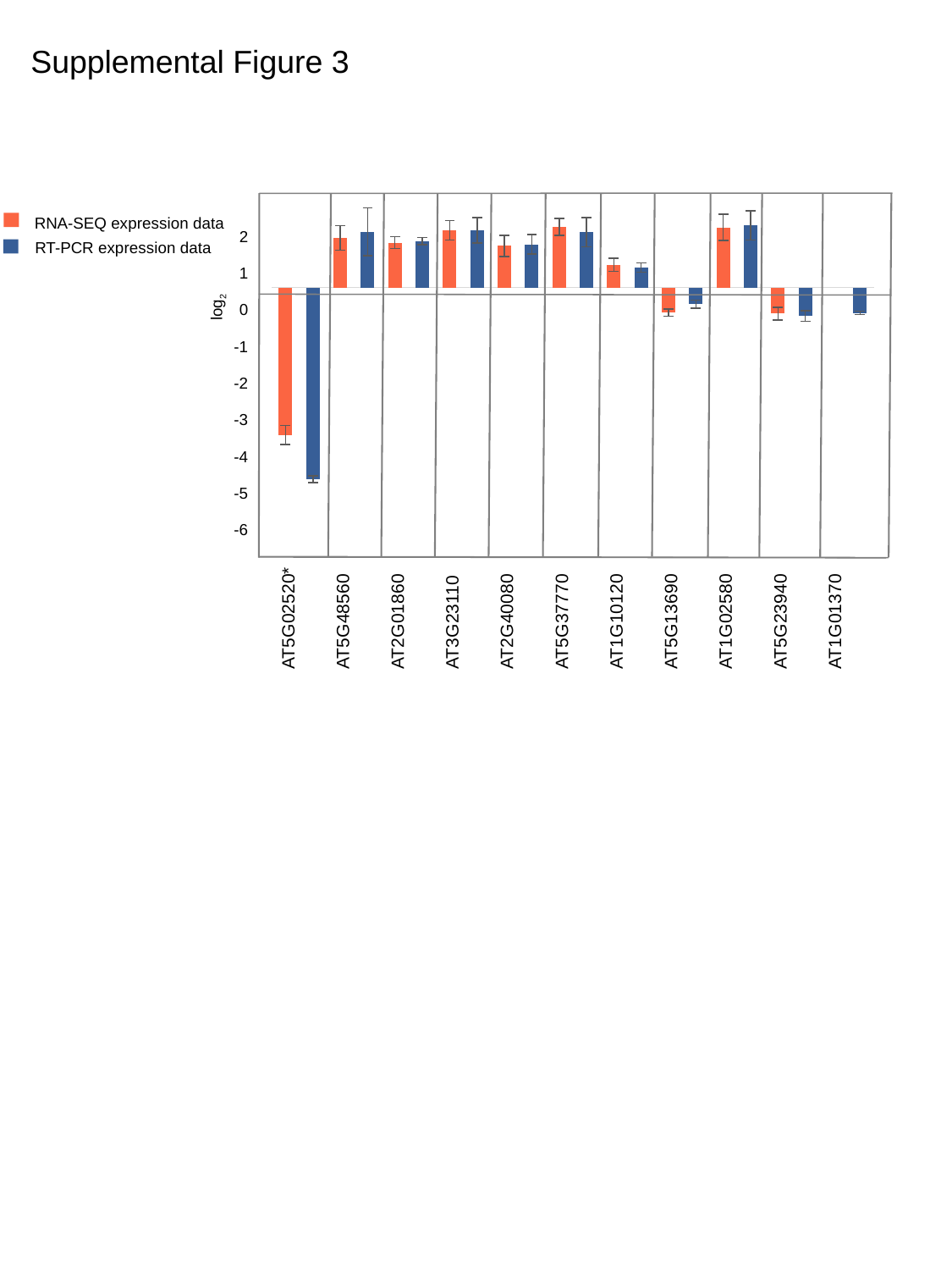

Supplemental Figure 3
2
1
0
-1
-2
-3
-4
-5
-6
### Chart
| Category | |
|---|---|
| AT5G02520 | -4.57490900825817 |
| | -5.9482610414623265 |
| AT5G48560 | 1.54430128477841 |
| | 1.7301065935151618 |
| AT2G01860 | 1.39532314966994 |
| | 1.4417335461862018 |
| AT3G23110 | 1.77647954821574 |
| | 1.7780958319347386 |
| AT2G40080 | 1.29665312149957 |
| | 1.3441192237518182 |
| AT5G37770 | 1.88178164362969 |
| | 1.7207144705445538 |
| AT1G10120 | 0.70315530819408 |
| | 0.6203137656825721 |
| AT5G13690 | -0.773389724467026 |
| | -0.5115293882151283 |
| AT1G02580 | 1.8651202432341 |
| | 1.927770231366041 |
| AT5G23940 | -0.804846828966577 |
| | -0.8811402444526847 |
| AT1G01370 | None |log2
AT5G02520*
AT5G48560
AT2G01860
AT3G23110
AT2G40080
AT5G37770
AT1G10120
AT5G13690
AT1G02580
AT5G23940
AT1G01370
RNA-SEQ expression data
RT-PCR expression data
