# Supplementary figures and images for: Depletion of KNL2 Results in Altered Expression of Genes Involved in Regulation of the Cell Cycle, Transcription, and Development in Arabidopsis
Source: Int J Mol Sci. 2019 Nov 15;20(22):5726. doi: 10.3390/ijms20225726 (PMC6888302; doi:10.3390/ijms20225726)

## Slide 1
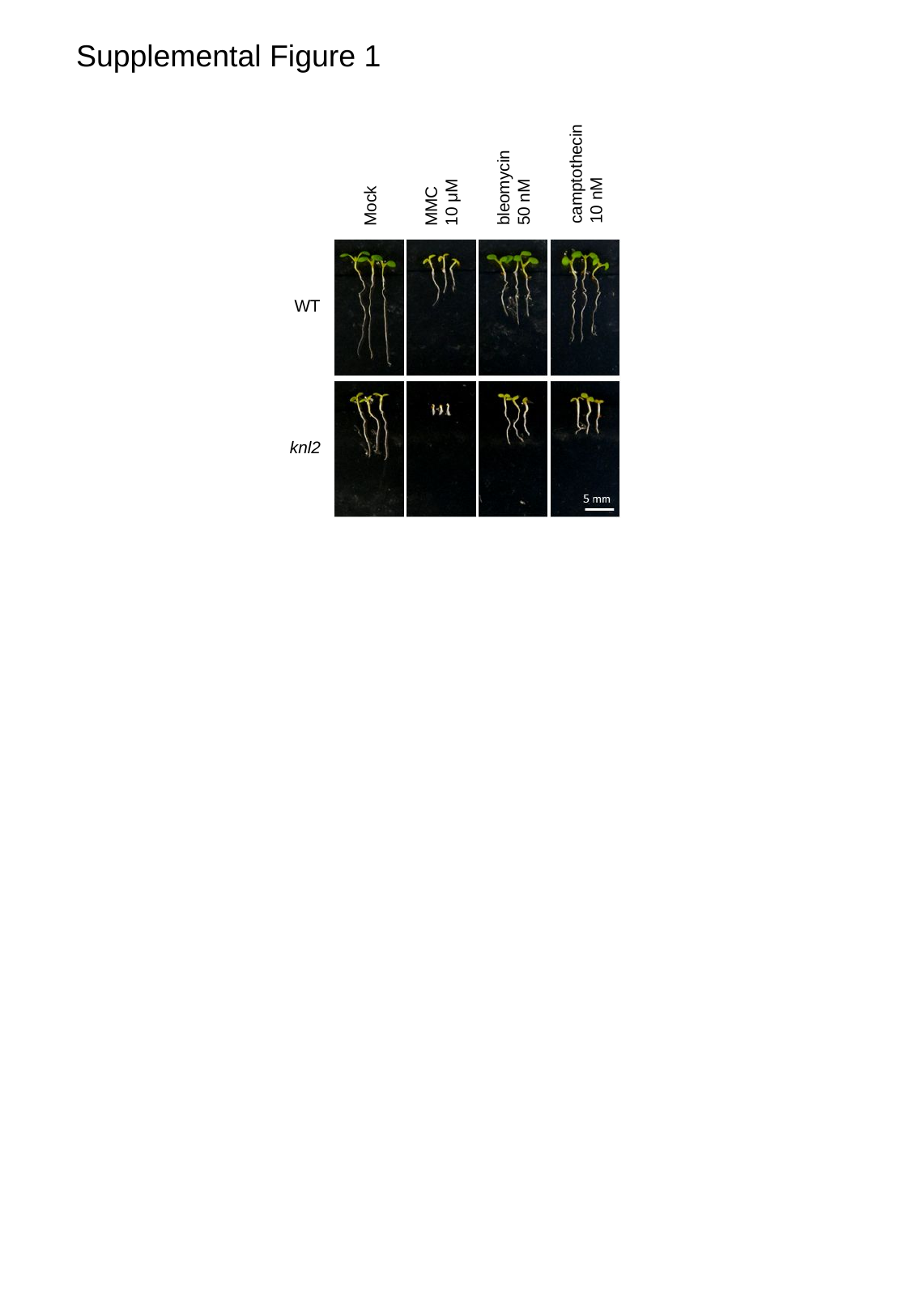

Supplemental Figure 1
camptothecin
10 nM
bleomycin
50 nM
MMC
10 μM
Mock
WT
knl2

Supplement: Supplementary file 1 [file ijms-20-05726-s001.zip › supplemental/Supplemental Figure 1.pptx]

## Slide 1
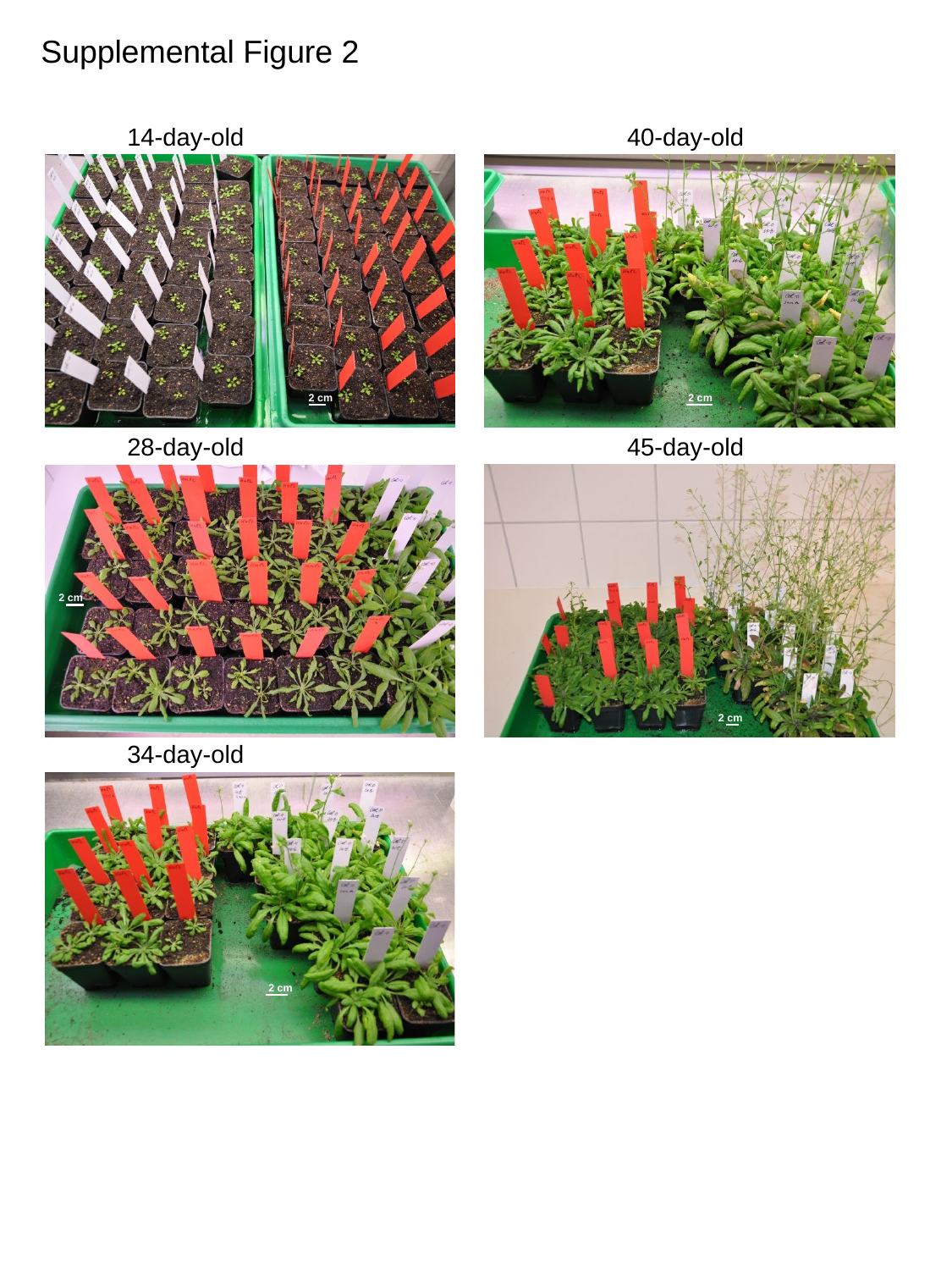

Supplemental Figure 2
14-day-old
40-day-old
2 cm
2 cm
28-day-old
45-day-old
2 cm
2 cm
34-day-old
2 cm

Supplement: Supplementary file 1 [file ijms-20-05726-s001.zip › supplemental/Supplemental Figure 2.pptx]
